# Multimodality Multiparametric Imaging of Early Tumor Response to a Novel Antiangiogenic Therapy Based on Anticalins

**DOI:** 10.1371/journal.pone.0094972

**Published:** 2014-05-06

**Authors:** Reinhard Meier, Rickmer Braren, Yvonne Kosanke, Johanna Bussemer, Frauke Neff, Moritz Wildgruber, Sarah Schwarzenböck, Annette Frank, Bernhard Haller, Andreas M. Hohlbaum, Markus Schwaiger, Hendrik Gille, Ernst J. Rummeny, Ambros J. Beer

**Affiliations:** 1 Department of Radiology, Klinikum Rechts der Isar, Technische Universität München, Munich, Germany; 2 Department of Nuclear Medicine, Klinikum Rechts der Isar, Technische Universität München, Munich, Germany; 3 Institute of Experimental Genetics, Helmholtz Zentrum München, Munich, Germany; 4 Institute for Medical Statistics and Epidemiology, Technische Universität München, Munich, Germany; 5 Pieris AG, Freising, Germany; Alexion Pharmaceuticals, United States of America

## Abstract

Anticalins are a novel class of targeted protein therapeutics. The PEGylated Anticalin Angiocal (PRS-050-PEG40) is directed against VEGF-A. The purpose of our study was to compare the performance of diffusion weighted imaging (DWI), dynamic contrast enhanced magnetic resonance imaging (DCE)-MRI and positron emission tomography with the tracer [^18^F]fluorodeoxyglucose (FDG-PET) for monitoring early response to antiangiogenic therapy with PRS-050-PEG40. 31 mice were implanted subcutaneously with A673 rhabdomyosarcoma xenografts and underwent DWI, DCE-MRI and FDG-PET before and 2 days after i.p. injection of PRS-050-PEG40 (n = 13), Avastin (n = 6) or PBS (n = 12). Tumor size was measured manually with a caliper. Imaging results were correlated with histopathology. In the results, the tumor size was not significantly different in the treatment groups when compared to the control group on day 2 after therapy onset (P = 0.09). In contrast the imaging modalities DWI, DCE-MRI and FDG-PET showed significant differences between the therapeutic compared to the control group as early as 2 days after therapy onset (P<0.001). There was a strong correlation of the early changes in DWI, DCE-MRI and FDG-PET at day 2 after therapy onset and the change in tumor size at the end of therapy (r = −0.58, 0.71 and 0.67 respectively). The imaging results were confirmed by histopathology, showing early necrosis and necroptosis in the tumors. Thus multimodality multiparametric imaging was able to predict therapeutic success of PRS-050-PEG40 and Avastin as early as 2 days after onset of therapy and thus promising for monitoring early response of antiangiogenic therapy.

## Introduction

Angiogenesis is one of the hallmarks of cancer biology as first described by J. Folkman 1971 [1]. Thus angiogenesis is an interesting target for anticancer therapy. While the first studies on antiangiogenic monotherapy have been disappointing, recently monotherapy with antiangiogenic multi-tyrosinekinase inhibitors such as Sorafinib has shown promising results in clinical trials with metastasized renal cell carcinoma [2]. Also the combination of cytotoxic and antiangiogenic therapy of FOLFIRI [3] with Avastin (Bevacizumab) [4] is now widely used.

Avastin is an antibody directed against the vascular endothelial growth factor -A (VEGF-A). VEGF is one of the key factors in the angiogenic cascade. When tumor growth exceeds approximately 2–3 mm^3^ the tumor becomes hypoxic leading to the expression of several hypoxia related genes. Tumors then start to produce a multitude of angiogenic factors such as VEGF which then diffuse towards nearby pre-existing blood vessels and bind to their specific receptors located on endothelial cells such as the receptors for VEGF (VEGFR-1/Flt-1, VEGFR-2/KDR/Flk-1, Nrp-1/neuropilin-1) [5], [6]. Receptor binding leads to receptor dimerization and trans-autophosphorylation on several tyrosine residues in the intracellular domain. The downstream activation of various signal transduction pathways, such as protein and lipid kinases, consequently leads to activation of endothelial cells by enhancing proliferation and migration [7]–[9]. Subsequently different mechanisms can lead to the formation of new blood vessels [10], [11].

The application of antibodies, such as Avastin, in antiangiogenic therapy has several disadvantages. Their composition demand complex manufacture and their Fc region lead to substantial side effects [12]. Bevacizumab has been shown to trigger thromboembolic complications in a subset of patients which are sometimes fatal [13]. The Bevacizumab Fc region has been implicated in these reactions via interaction of this domain with the platelet FcgammaRIIa [14]. Moreover the relatively large size of antibodies causes pharmacokinetic disadvantages like impaired diffusion into dense tumors. Thus smaller structures targeting angiogenic factors might be advantageous as pharmaceutical agents. Anticalins are a novel class of targeted protein therapeutics based on the human lipocalin protein scaffold. Due to its relatively small size the PEGylated Anticalin Angiocal (PRS-050-PEG40) might be an interesting alternative to currently used VEGF-targeted antibodies.

Usually only a subset of patients responds to antiangiogenic targeted therapy. Therefore it is of great clinical relevance to stratify these responders from non-responders either before or at an early time point after start of therapy. Molecular imaging lends itself for this purpose as it is non-invasive and can cover large areas of the body in case of metastatic disease. Positron emission tomography (PET) using ^18^F-fluoro-deoxy-glucose (FDG), but also dynamic contrast enhanced magnetic resonance imaging (DCE-MRI) and diffusion weighted magnetic resonance imaging (DWI) are increasingly used imaging techniques for response assessment. While FDG-PET assesses the effects of therapy by evaluation of the glucose metabolism of tumors, DCE-MRI characterizes perfusion as a potential surrogate parameter of angiogenesis and DWI measures water movement potentially reflecting tissue cellularity. All these imaging modalities are already being used in the clinic for the assessment of tumor biology and therapy response [15]. However it is not yet known whether response assessment of Anticalin based therapy using these imaging biomarkers is feasible. Thus in this study we investigated for the first time the feasibility of early response assessment to a novel antiangiogenic therapy using the PEGylated Anticalin Angiocal (PRS-050-PEG40) with DWI, DCE-MRI and FDG-PET in a preclinical sarcoma model and compared it to the activity of Avastin in this model.

## Materials and Methods

### Animal model and study protocol

This study was approved by the ethics committee on animal research of the government of Upper Bavaria, Germany. Animals were anesthetized by gaseous infusion of isofluorane at 1.5% (Abbott GmbH, Wiesbaden, Germany) for tumor implantation and for MR imaging and monitored using a small animal monitoring system (SA Instruments Inc., NY, USA). Thirty-one 6–8 week old female mice (swiss nu nu, Charles River, Wilmington, MA) were implanted subcutaneously A673 rhabdomyosarcoma xenografts by subcutaneous injection of 3,5×10^6^ A673 cells into the lower lateral right flank of the mice. Tumor size was measured manually with a caliper. When the tumors reached a size of approximately 3–5 cm^3^ they underwent DWI, DCE-MRI and FDG-PET before (pre) and 2 days after (post) i.p. injection of 100 µL of PRS-050-PEG40 (15 mg/kg) (n = 13), Avastin (5 mg/kg) (n = 6) or PBS (n = 12). Due to technical difficulties and early death, a reduced number of valid DCE-MRI datasets was available at pre and post (PRS-050-PEG40 (n = 7), Avastin (n = 3) or PBS (n = 8)). The animals were sacrificed at day 7 after start of therapy, subsequently histological analyses of the tumor tissue were performed. For histological correlation an additional group of mice with subcutaneously A673 rhabdomyosarcoma xenografts were sacrificed before (n = 3) and 2 days after injection of PRS-050-PEG40 (n = 3), Avastin (n = 4) and PBS (n = 3).

### Therapy

Anticalin are a novel class of targeted protein therapeutics based on the human lipocalin protein scaffold. These lipocalins endogenously bind, store and transport a broad spectrum of molecules [16]. Lipocalins are monomeric and stable single β-barrel domain proteins (18–20 kDa), which are present in blood plasma and body fluids and non-immunogenic [16]. The PRS-050 Anticalin is based on human tear lipocalin and was derived from a combinatorial phage display library in which the four loop regions which connect the beta sheets at the entrance to the natural binding pocket had been randomized. Panning and screening campaigns against human VEGF-A (residues 8–109) were performed. A free cysteine was introduced into the final candidate by site-directed mutagenesis and this residue was subjected to site-directed PEGylation using a 40 kDa poly-ethylene-glycol maleimide in order to reduce renal clearance in vivo. The detailed generation and initial characterization of this molecule will be described elsewhere. The PEGylated Anticalin Angiocal (PRS-050-PEG40) has shown good efficacy in a s.c. A673 and other xenograft models in previous studies [17].

### Magnetic resonance imaging

MRI scans were performed on a 1.5 T clinical MR system (1.5T Achieva, Philips Medical Systems, Best, The Netherlands). After fixation of a tail vein catheter animals were placed in prone position onto a 47-mm microscopy surface coil (Philips Medical Systems, Best, The Netherlands). Following a survey scan, a multi-slice T2-weighted turbo spinecho sequence, covering the tumor region, was applied as anatomical reference, for tumor detection and tumor volume quantification (slice thickness = 0.7 mm, in plane resolution = 0.3×0.3 mm^2^, TR/TE = 3170/90 ms, number of signal averages (NSA) = 8).

Subsequently a transverse multi-slice diffusion-weighted MRI (DWI) sequence covering the tumors was performed (in plane resolution = 0.3×0.3 mm^2^, TSE factor = 43, TR/TE = 2500/58 ms, b_0–2_ values = 20, 200, 600 s/mm^2^, NSA = 10). ADC values were calculated in the Interactive Data Language (ITT VIS, Boulder, CO, USA). For DWI analyses, ROIs were manually defined in the ADC map. A mean ADC value was calculated for each tumor from all slices analyzed excluding slices exhibiting severe distortion artifacts.

DCE-MRI studies were performed using a previously described fast single-shot look-locker-based radial T1 mapping technique [18]–[20]. Dynamic T1 mapping of an transverse slice of the center region of the tumor was performed every 6 sec during the first 3 min and thereafter every 24 sec until 15 min (radial profiles = 225, flip angle = 10°, T_acq_ = 2.9 sec, T_pause_ = 3.1 sec, slice thickness = 2 mm, field of view = 80×80 mm^2^, matrix size = 100×100 pixel, TR/TE = 11.6/5 msec, NSA = 1). Gadolinium diethylenetriamine penta-acetic acid (Gd-DTPA) (0.2 mmol Gd kg^−1^, Magnevist®, Bayer Schering, Berlin, Germany) was manually injected as a bolus after 60 sec. ROIs were drawn manually in tumor tissue and spinal muscle in order to derive mean T1 values. ROIs of the tumor tissue covered the central part of the tumors regardless of present necrosis. The resulting mean T1 time curves were converted to Gd-DTPA concentration (CGd) time curves assuming a linear relationship between tissue relaxation rate R1 (1/T1) and CGd [21]: R_10_ = R_10_ + r1CGd with r1 (relaxivity of Gd-DTPA) = 4.1 sec^−1^ mM^−1^. R10 (1/T_10_) is the relaxation rate of the respective tissue without Gd-DTPA as determined from the averaged tissue T1 values prior to contrast agent injection.

### PET

For FDG-PET imaging animals were routinely fasted 4 hrs prior to imaging with μPET (Inveon, SIEMENS Preclinical Solutions, Knoxville, TN). Static image acquisition was performed 45 min after injection of 5–10 MBq [^18^F]-FDG for 15 min. Data was reconstructed with a filtered backprojection algorithm with a cut-off at the Nyquist frequency. Image data was corrected for normalization, dead time and decay. No corrections for scatter and attenuation were carried out. Large 3-dimensional volumes of interest (VOI) covering the entire tumor volume were drawn in the PET images. Threshold VOIs were then defined to select the maximum 50% count rates, which were considered to reflect the vital tumor mass. Two 2-D ROIs were placed in muscle to determine background uptake. Tumor to muscle (T/M) ratios were calculated.

### Data analysis and statistics

Tumor size was measured manually with a caliper. DCE-MRI and DWI data were analyzed using in-house software written in IDL. Quantitative data are presented as mean and standard deviation (mean±standard deviation), for qualitative data absolute and relative frequencies are shown. For comparison of quantitative measures of pre and post data between the three study groups a closed testing procedure was applied. First an analysis of variance (ANOVA) comparing means of all groups was performed on a two-sided level of significance of 5%. If the null hypothesis could be rejected, pairwise two-sample t tests were conducted on a 5% level of significance. Differences of group means derived from the pairwise comparisons with 95% confidence intervals are presented. In order to assess associations between tumor growth measurements and imaging modalities and associations between results of different imaging devices Pearson's correlation coefficients were estimated. All statistical computations were processed using SPSS 15.0. (SPSS Inc, Chicago, IL).

### Histopathology

Tumors were fixed in 4% neutral buffered formalin for 24–48 h at 4°C, dehydrated under standard conditions, and embedded in paraffin. Serial four µm-thin sections were collected every 500 µm and subjected to hematoxilin and eosin (H&E) staining for quantitative histological analysis. Furthermore, to detect the microvessel density (MVD), immunohistochemistry for smooth muscle antigen (SMA) and endothelial CD31 antigen (both abcam, Cambridge, UK) was performed using the Discovery®XT autostainer from Roche-Ventana (Penzberg, Germany). Images were taken using a virtual imaging system, the Olympus dotslide 2.0 (Olympus New Zealand PTY LTD, Auckland, New Zealand) or the Hamamatsu® Nanozoomer HT (Hamamatsu Photonics, Herrsching, Germany). The total tumor and necrosis area were measured using the NDPview® software from Hamamatsu. A pathologist who was blinded to the imaging data determined the histological results and rated the microvessel density (MVD) on CD31 and SMA immunohistochemistry in a semiquantitative scoring system adapted from the “dako-score” using a four-point scale: 0 = very weak, 1 = weak, 2 = moderate and 3 = intense staining.

## Results

### Tumor size measurements

**Figure 1:**
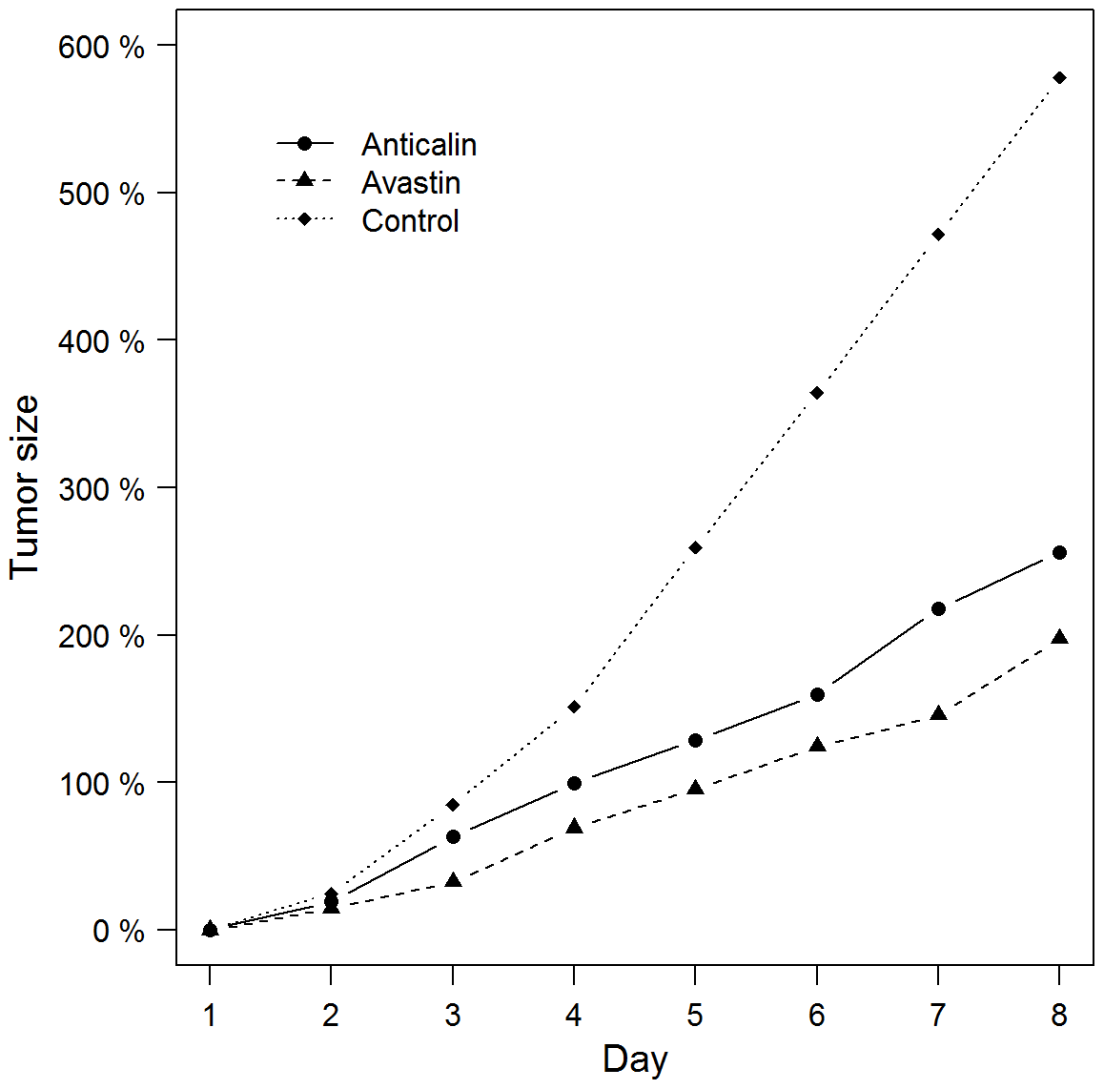
Percentage change of the tumor size. On day 3 (day 2 after therapy onset) tumor volume between the different groups did not reach a statistical significance (PRS-050-PEG40/Avastin/PBS, P = 0.09). On day 8 (day 7 after therapy onset) tumors in the control group were significantly larger compared to the treatment groups (PRS-050-PEG40/Avastin in comparison to PBS, each P = 0.001). There was no significant difference in the tumor growth between the therapy groups PRS-050-PEG40 and Avastin neither on day 2 nor day 7 after therapy onset (P = 0.13/0.30). Data are mean values.

**Figure 2:**
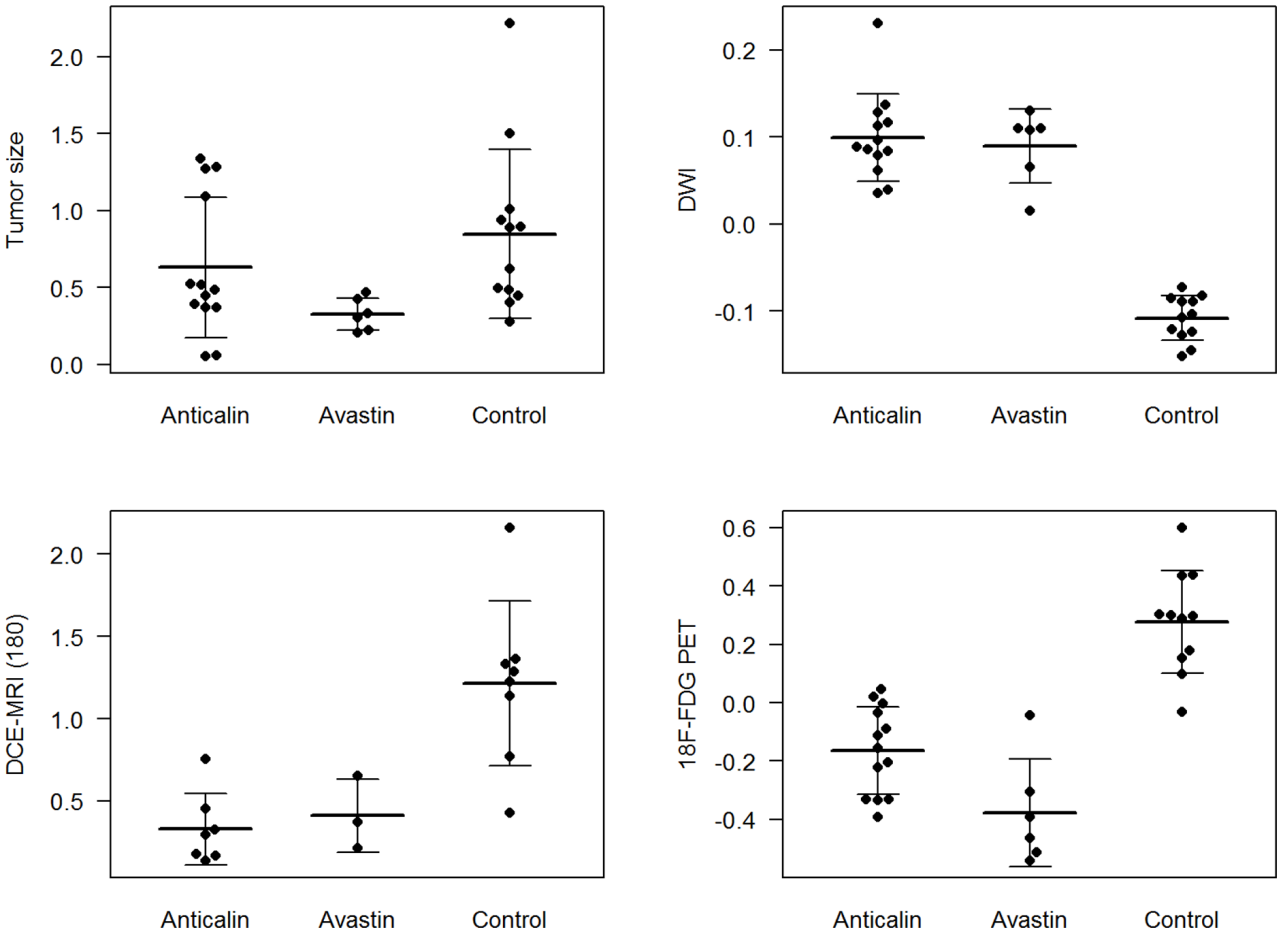
Percentage change (1.0 = 100%) of all subjects (mean and standard deviation depicted as horizontal and vertical line) of the tumor size, DWI, DCE-MRI and FDG-PET on day 2 after onset of therapy (PRS-050-PEG40 or Avastin) or injection of PBS as control group.

Baseline tumor volumes at day 1 were not significant different between the different groups Anticalin PRS-050-PEG40 (337.6±203.1 mm^2^), Avastin (556.5±173.3 mm^2^) or PBS (531.7±641.8 mm^2^)(P = 0.44). There was a tumor growth notable on day 2 after therapy onset (post therapy, p.t.). However, the differences of the tumor growth between the different groups did not reach a statistical significance at this early time point (Anticalin, Avastin, PBS: means and standard deviations: 63.1±45.5%, 32.6±10.6%, 84.8±55.0%; P = 0.09) (Figure 1, Figure 2). Tumors were allowed to grow up to 8 days depending on tumor size. Then animals had to be sacrificed due to the large tumor size in the placebo group. At this late time point, 7 days after therapy start, tumors in the placebo group were significantly larger compared to the treatment groups (Anticalin/Avastin in comparison to PBS: 256.1±170.5%/197.7±56.9% in comparison to 578.2±284.0%, each P = 0.001) (Figure 1). There was no significant difference in the tumor growth between the therapy groups Anticalin and Avastin neither on day 2 nor day 7 after therapy onset (Anticalin in comparison to Avastin day 2/7 p.t., P = 0.13/0.30) (Figure 1).

### Multiparametric imaging

**Figure 3:**
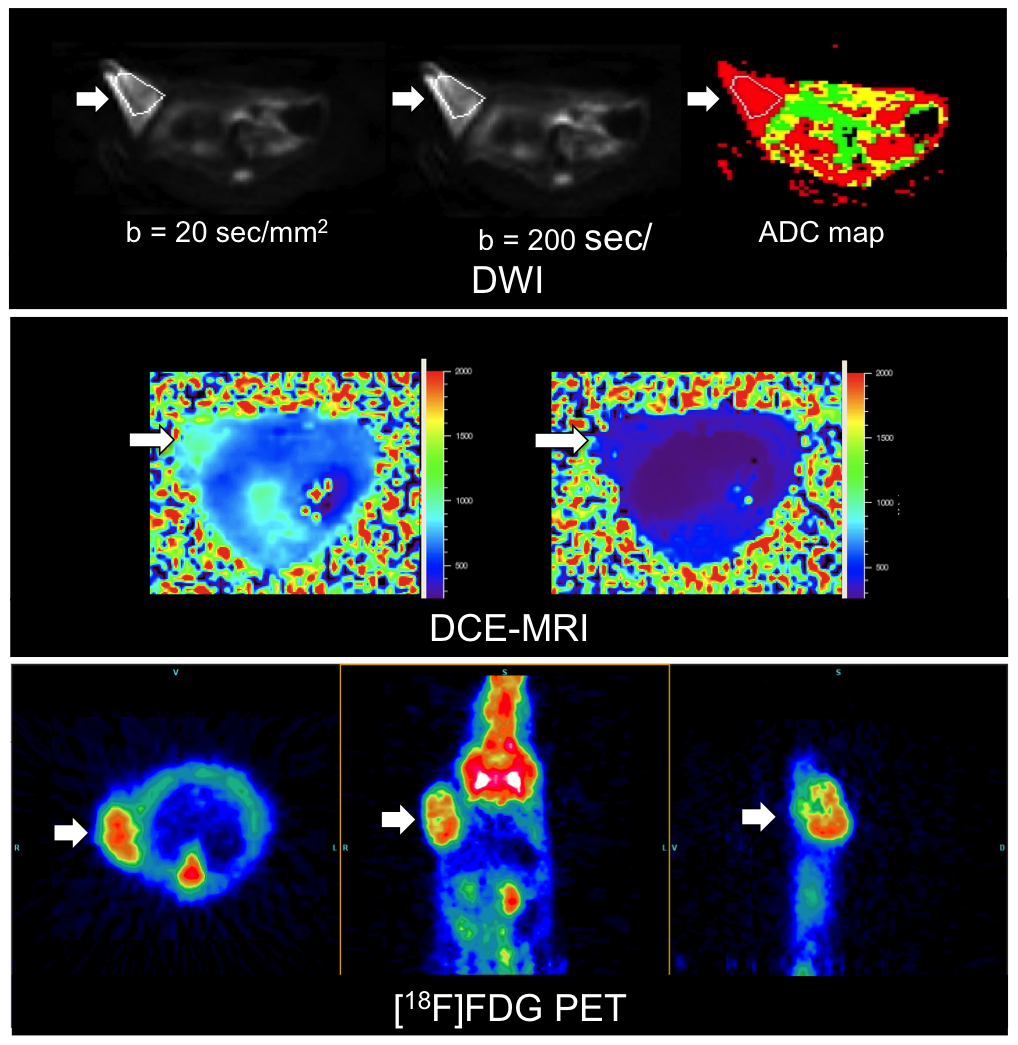
Representative DWI (transverse slices; b = 20, b = 200, ADC map), T1 map (T1 values are shown in color scale (ms)) of DCE-MRI from a single time point (transverse slices; pre and post i.v. injection of Gadolinium) used for T1 quantification and FDG-PET (from left to right: transverse, coronal and sagittal reconstruction) images of the A673 rhabdomyosarcoma xenografts (arrows) subcutaneously implanted in the right lateral flank.

**Figure 4:**
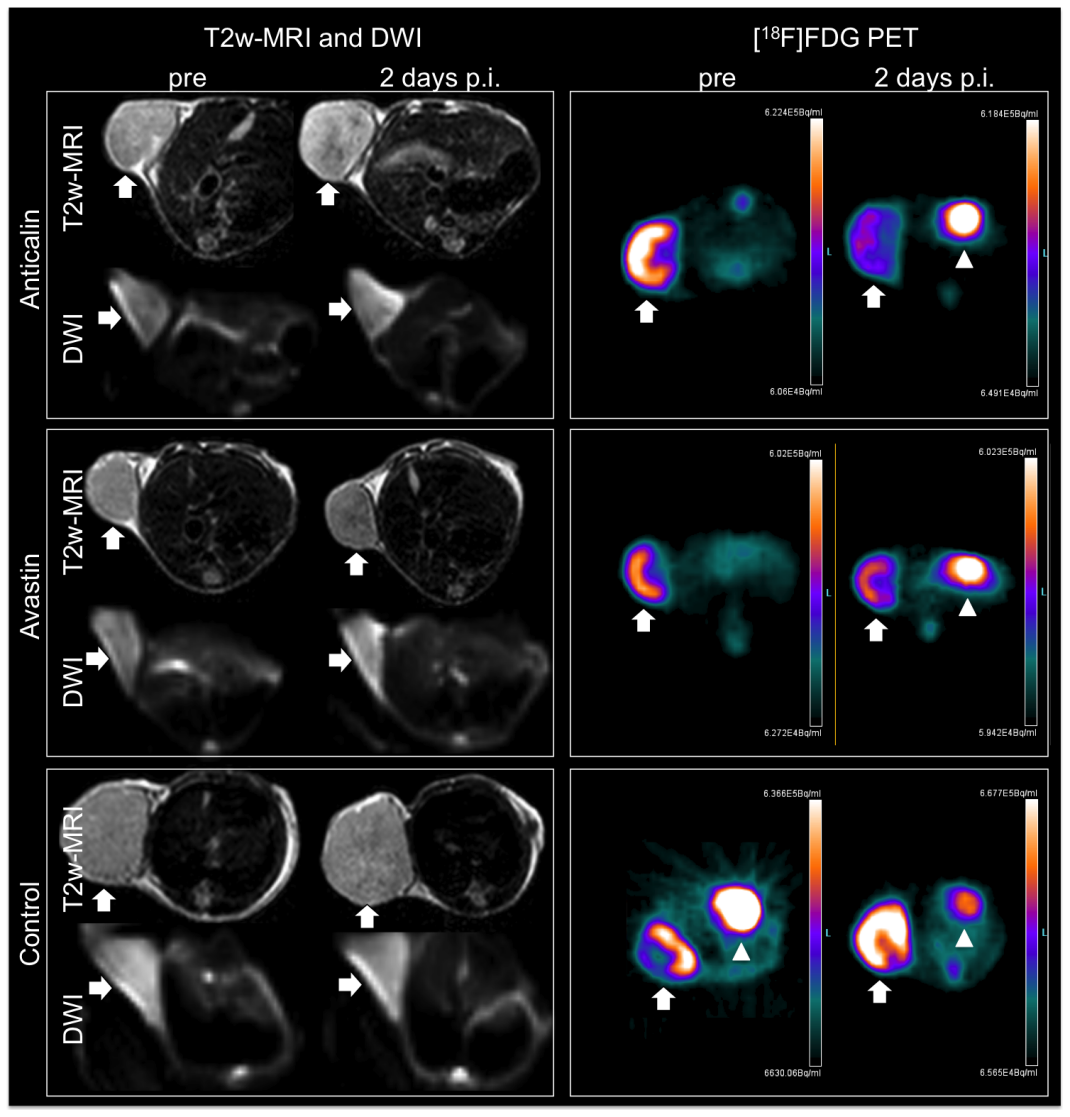
Representative transverse slices of T2w-MRI, DWI and FDG-PET of the subcutaneously implanted A673 rhabdomyosarcoma xenografts (arrows) pre and 2 days after therapy onset (p.i.) with injection of PRS-050-PEG40, Avastin or PBS as control group. On T2-weighted MR images the treated tumors showed discreet central hypointense changes indicating apoptosis while the control group did not show significant signal alterations of the tumor tissue. DWI (b = 200) of the PRS-050-PEG40 and Avastin treated tumors shows a small increase in signal intensity while the control group shows a discreet decrease in concordance with the ADC value. FDG-PET demonstrates a decrease in FDG uptake (radioactivity is shown in color scale (Bq/mL)) in the PRS-050-PEG40 and Avastin treated tumor tissue, while the control tumor shows an increase in FDG uptake. Of note, the PET images reveal physiologic cardiac FDG uptake (arrowheads).

T2-weighted MRI, DWI and DCE-MRI allowed the identification of tumors, located subcutaneously in the right flank of the mice at all imaging time points (Figure 3, Figure 4). On T2-weighted MR images the treated tumors showed discreet central hypointense changes while the control group did not show significant signal alterations of the tumor tissue on day 2 after therapy onset. In DWI the therapeutic groups showed a significant increase in ADC value (Anticalin: 10.0±5.0%, Avastin: 9.0±4.2%), while the control group showed a decrease (−10.8±2.6%) (PRS-050-PEG40/Avastin in comparison to PBS, each P<0.001) on day 2 after therapy onset (Figure 2). There was no significant difference in the ADC value between the therapy groups Anticalin PRS-050-PEG40 and Avastin on day 2 after therapy onset (Anticalin vs. Avastin, P = 0.67). DCE-MRI showed a significantly lower increase in the IAUC180 value in the therapeutic groups (Anticalin: 33.0±21.7%, Avastin: 41.2±22.2%) compared to the control group (PBS: 121.3±50.0%)(Anticalin/Avastin in comparison to PBS, each P = 0.001) on day 2 after therapy onset. There was no significant difference in the IAUC180 value between the therapy groups Anticalin PRS-050-PEG40 and Avastin on day 2 after therapy onset (Anticalin vs. Avastin P = 0.62). Furthermore there was no significant difference of the IAUC120 or IAUC90 value between the groups (P = 0.75, P = 0.72), most probably due to the inhomogeneity of the tumor tissue as well as artifacts resulting from the subcutaneous tumor model and motion of the animal. The FDG-PET Signal decreased significantly in the therapeutic groups (Anticalin: −16.4±15.0%, Avastin: −37.6±18.5%), whereas there was an increase in FDG signal in the control group on day 2 after therapy onset (27.8±17.6%) (Anticalin/Avastin in comparison to PBS each P<0.001). There was no significant difference in the FDG signal between the therapy groups Anticalin and Avastin on day 2 after therapy onset (P = 0.39).

**Figure 5:**
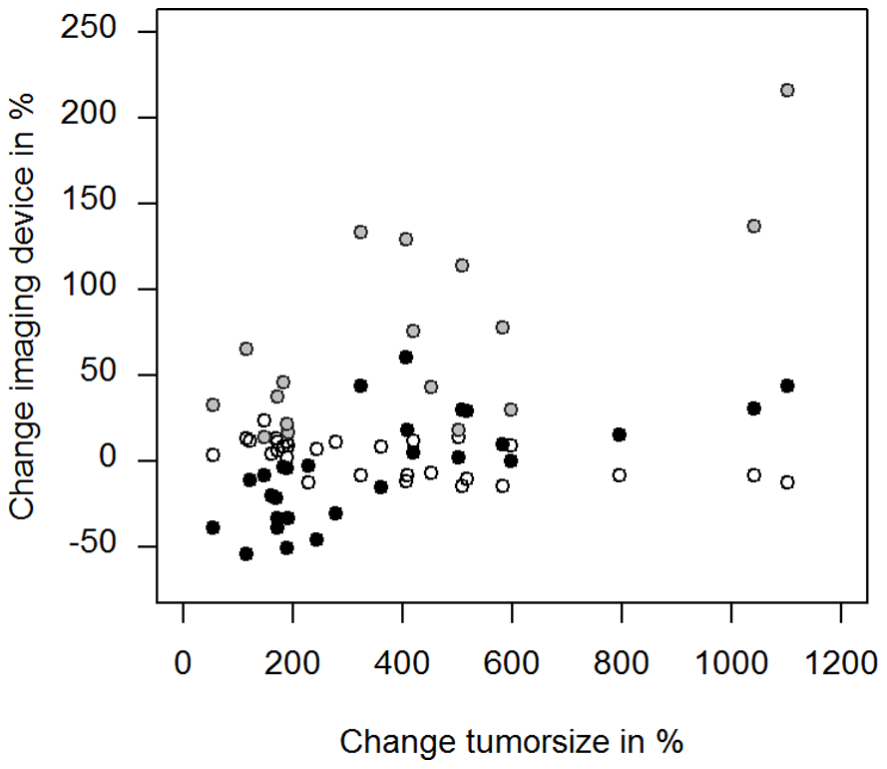
Scatter plots illustrating the degree of correlation between the percentage change of tumor size (pre vs. day 7 after onset of therapy) plotted along the horizontal axis versus DWI (hollow circle, r = −0.58, P = 0.001), DCE-MRI (grey circle, r = 0.71, P = 0.001) and FDG-PET (black circle, r = 0.67, P<0.001) (pre vs. day 2 after onset of therapy) plotted along the vertical axis.

The correlation coefficient ‘r’ of the percentage change of the tumor size (pre vs. day 7 after onset of therapy) versus DWI, DCE-MRI (IAUC180) and FDG-PET was −0.58 (P = 0.001), 0.71 (P = 0.001) and 0.67 (P<0.001) respectively (Figure 5). It has to be noted however, that the DCE-MRI group was dominated by one outlier. Thus the results concerning DCE-MRI still have to be interpreted with caution. The correlation coefficient ‘r’ of the percentage change of DCE-MRI (IAUC180) versus DWI, DWI versus FDG-PET and DCE-MRI (IAUC180) versus FDG-PET was −0.76, −0.69 and 0.75 respectively (P<0.001, each).

### Histopathology

**Figure 6:**
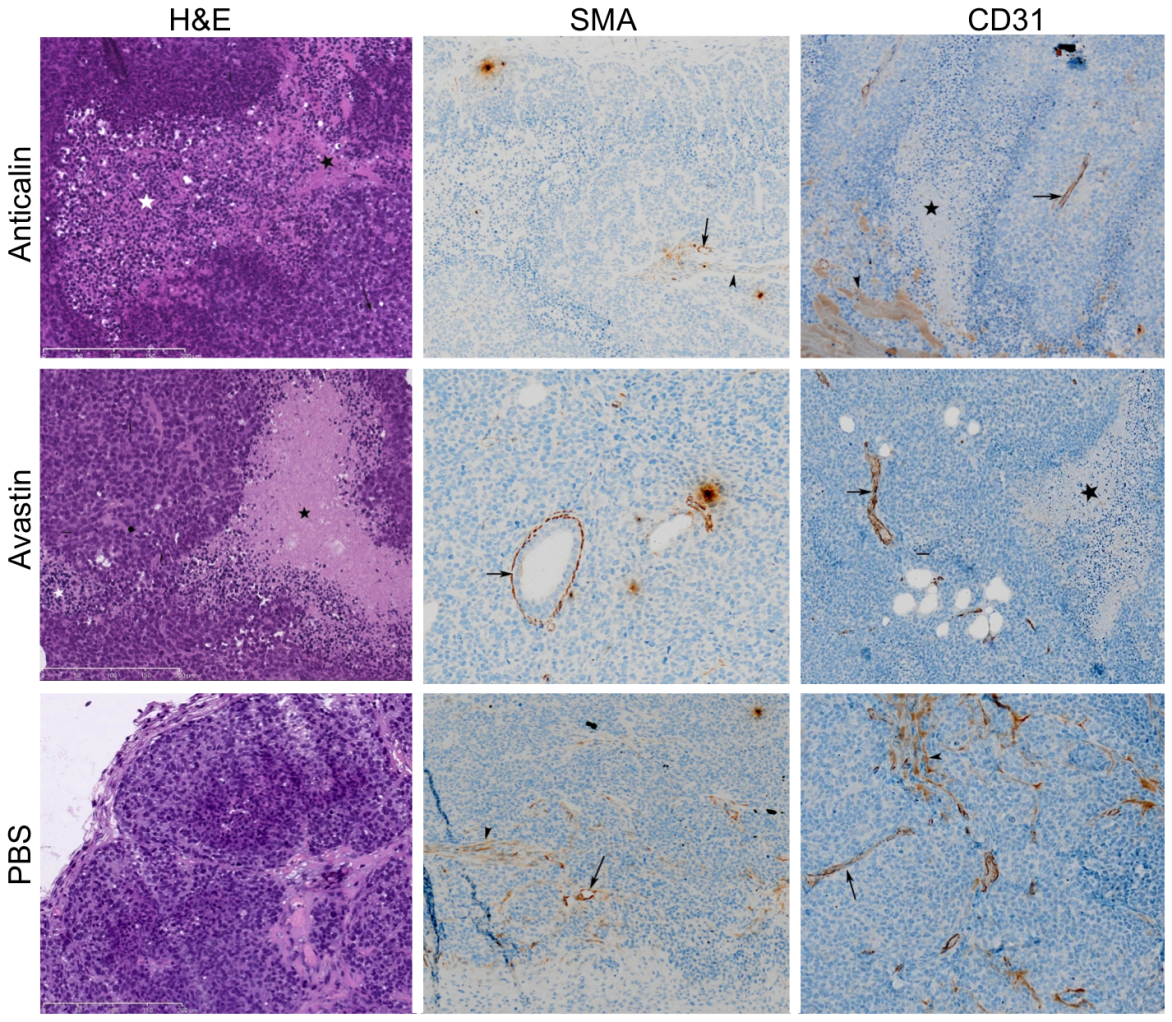
Representative H&E, SMA and CD31 stains of PRS-050-PEG40, Avastin or PBS treated tumors at day 2 after onset of therapy. The representative Anticalin treated tumor shows extensive necrosis (black star) and necroptosis (white star), the Avastin treated tumors shows extensive necrosis and a mitosis (block dot). In contrary the PBS treated control tumor shows no necrosis or apoptosis. The black arrows indicate vessels, black arrowheads indicate musculature and black dash indicates apoptosis.

H&E, SMA and CD31 stains of the Anticalin PRS-050-PEG40 treated tumors (2 days p.t. (n = 3), 7 days p.t. (n = 4)) showed predominantly apoptosis and necroptosis, but rarely necrosis at day 2 after onset of therapy. The Avastin treated tumors (2 days p.t. (n = 4), 7 days p.t. (n = 6)) showed huge necrosis and necroptosis, some apoptosis. In contrary, the PBS treated control tumors (pre (n = 3), 2 days p.t. (n = 3), 7 days p.t. (n = 3)) showed minor necrosis and apoptosis but much mitosis at this early time point (Figure 6). Necrosis was defined as complete cell fragmentation with oncosis and dismantling, as a trigger of inflammatory processes. Apoptosis was defined by an ensemble of morphological features, including chromatin condensation and nuclear fragmentation, cell shrinkage and plasma membrane blebbing. Necroptosis was defined as a combination of necrosis and apoptosis: extensive formation of apoptotic bodies, activation of cleaved caspase-3, linked to rapid mitochondrial dysfunction that leads to the excessive production of reactive oxygen species; however it is no trigger of inflammatory processes [22].

The semiquantitative analysis of the microvessel density (MVD) was assessed by CD31 and SMA immunostaining and revealed a major decrease of the MVD in the Anticalin PRS-050-PEG40 treated tumors (CD31: 2 days p.t. = 1.33±0.58, 7 days p.t. = 0.5±0.58; SMA: 2 days p.t. = 1.0±1.0, 7 days p.t. = 0.5±0.58) and the Avastin treated tumors (CD31: 2 days p.t. = 1.5±1.0, 7 days p.t. = 1.33±0.52; SMA: 2 days p.t. = 1.5±1.0, 7 days p.t. = 1.17±0.98). In contrary, the PBS-treated control group showed only a minor decrease of MVD (CD31: pre = 2.0±1.0, 2 days p.t. = 2.0±0, 7 days p.t. = 1.67±0.58; SMA: pre = 1.67±0.58, 2 days p.t. = 1.67, 7 days p.t. = 1.33±0.58)(Table 1).

**Table 1:** Semiquantitative analysis of the microvessel density (MVD) as assessed by CD31 and SMA staining.

	CD31			SMA			necrotic areas in %		
	before	2days p.t.	8 days p.t.	before	2days p.t.	8 days p.t.	before	2days p.t.	8 days p.t.
Avastin	/	1,50	1,33	/	1,50	1,17	/	16,62	30,91
Anticalin	/	1,33	0,50	/	1,00	0,50	/	7,21	55,17
Control	2,00	2,00	1,67	1,67	1,67	1,33	2,28	6,26	44,79

Quantification (%) of the necrotic areas in the tumor tissue before and 2 or 7 days post therapy (p.t.).

The quantification of the necrotic areas in the tumor tissue showed a major percentage increase of necrotic areas at 7 days after onset of therapy in the therapeutic as well as control groups (Anticalin: 2 days p.t. therapy = 7.21±5.2%, 7 days p.t. = 55.17±32.4%; Avastin: 2 days p.t. = 16.62±9.7%, 7 days p.t. = 30.91±12.8%; Control: pre = 2.28±2.4%, 2 days p.t. = 6.26±1.3%, 7 days p.t. = 44.79±9.9%).

## Discussion

In this study we showed that early response assessment to Anticalin based antiangiogenic therapy is feasible using multiparametric molecular imaging in a preclinical sarcoma model. Significant changes in the therapy versus control group could be seen using multimodality multiparametric imaging as early as two days after treatment and preceded volumetric changes in tumor growth. The results were robust for DCE-MRI, DWI and FDG-PET, as they visualized tumor cell death early after onset of therapy. Moreover, these early changes in imaging signals also showed a strong correlation with late changes in tumor size on an individual basis and thus were able to predict therapy success.

Our results did not reveal significant better antitumor effects of the novel drug Anticalin PRS-050-PEG40 as compared to Avastin. However the activity of the Anticalin PRS-050-PEG40 in the model was at least as effective as Avastin impacting all parameters used in this study to assess antiangiogenic responses at early and late time points. Thus PRS-050-PEG40 might be an alternative to Avastin since it is monovalent and lacks an Fc effector domain thus avoiding the formation of higher-order immune complexes and interactions with endogenous Fc receptors, respectively. PRS-050-PEG40 may also reach a higher local concentration in tumor tissue due to smaller size [16]. Thus Anticalins may be promising candidates for therapeutic applications. PRS-050 has demonstrated anti-tumor activity in several preclinical models, furthermore PRS-050 has successfully completed safety evaluation in a phase I patient setting [23,23].

To date, clinical trials with cytotoxic chemotherapeutic agents are mostly using bi-dimensional measurements (e.g. RECIST criteria) of the tumor extension in comparison with a baseline measure in order to estimate changes in response to the investigational therapy. However, antiangiogenic agents rather lead to a stop of tumor progression than to massive tumor shrinkage by changes in parameters such as tissue cellularity, blood flow or glucose metabolism [24]. Therefore, there is great interest in reliable biomarkers of early tumor response such as provided by the multiparametric imaging techniques presented in this study.

FDG-PET is an established clinical imaging method for assessing the effects of therapy objectively and quantitatively by evaluation of the glucose metabolism of tumors [25]. It has been demonstrated in different clinical studies that changes of FDG uptake after therapy significantly correlate with histopathologic response and survival of patients, thus FDG PET has a prognostic value for therapy assessment of different kinds of cancer [26]–[29]. Our study is in line with the cited earlier reports, as FDG-PET showed significant changes in tumor metabolism as early as 2 days after therapy onset allowing for early therapy response evaluation. In the treated tumors we saw a slowdown of the tumor growth, correlating to histologic apoptosis and necroptosis, while the control group showed an increase in tumor metabolism correlating to further tumor growth. Of note, FDG-PET was the imaging method with the highest correlation to Δ tumor volume day1/8 in this study. However, we encountered in FDG-PET, as well as in DWI and DCE-MRI, variability in signal and parameter values between the animals most probably due to different tumor sizes.

Recently, DWI has been successfully introduced as an imaging tool in oncology and as a cancer biomarker [30], [31]. It is clinically established, easy to use and not invasive [31]. The apparent diffusion coefficient (ADC), reflecting restricted tissue water movement, can be successfully used for longitudinal monitoring of tumor response to vascular disrupting agents even in preclinical tumor models [32], [33]. Our results are in accordance with these earlier studies. The ADC values showed a significant change as early as 2 days after therapy onset allowing for early therapy response evaluation. The antiangiogenic therapy resulted in an increase of ADC value 2 days after onset of therapy paralleled by progressive histologic apoptosis and necroptosis; in contrary the control group showed a significant decrease in ADC value 2 days after onset of therapy corresponding to further tumor growth. Moreover, early changes in ADC correlated strongly with tumor size changes at the end of therapy. Furthermore DWI offers good correlation with FDG-PET, the imaging method with the highest correlation to Δ tumor volume day 1/8 in this study. The increase of the ADC value in the therapeutic group with antiangiogenic therapy can be explained by overlapping biological phenomena responsible for the signal of DWI and FDG-PET: both are influenced by the number and density of viable tumor cells. Histopathology revealed early apoptosis and necroptosis in the tissue samples in the treatment group and thus less viable tumor cells and less restricted water movement. This may explain the correlated changes for both imaging parameters.

DCE-MRI has also evolved as a promising technique for the characterization of tumor viability [34] and monitoring of antiangiogenic therapies in clinical studies [35]–[38]. In our study we used a radial T1 mapping method for the acquisition of contrast agent dynamics [39] instead of a commonly used dynamic T1-weighted gradient echo sequence [40]. Advantages of this method are its low susceptibility to motion and flow artifacts, thus animals can be imaged under free breathing condition and analyzed using a reference region (RR) based PK model [18], [41]. We calculated IAUC values which are easy to calculate, reasonably reproducible and are routinely used as a biomarker in drugs trials [42]. We did not perform the common calculation of the tissue transfer constant K^trans^ and the extravascular/-cellular volume fraction v_e_ computed from one or two compartment PK models because it is based on complex calculations and idealized estimations which limits its application in clinical trials [43]. In accordance to other publications [41], [44] we could demonstrate a lower increase in perfusion in the therapy group compared to the control group using the IAUC180 value at 2 days after therapy onset. Furthermore, DCE-MRI shows strong correlation to Δ tumor volume day1/8 and it shows strong correlation to the other imaging methods DWI and FDG-PET. However, there was no significant difference of the IAUC120 or IAUC90 value between the groups (P = 0.75, P = 0.72). Thus the results concerning early response evaluation with DCE-MRI were less robust and conclusive in our study as compared to DWI and FDG-PET. This result is surprising as measurement of tissue perfusion should be ideally suited for assessment of response to antiangiogenic therapy. Also histopathology revealed a decrease in microvessel density as assessed by CD31 staining. One limiting factor might be the use of a clinical scanner for small animal imaging instead of a dedicated high-field small animal MRI system. However, also clinically the results of DCE-MRI for assessment of response to antiangiogenic therapy are heterogeneous and sometimes disappointing and strongly depend on the specific tumor type and therapy regimen under investigation [31].

We recognize several limitations of our study. Our *in vivo* investigations were obtained in a limited number of rodents concerning DCE-MRI studies. In addition we used a clinical MR scanner with limited spatial resolution. Thus a voxelwise analysis of imaging parameters was not possible. Furthermore we encountered susceptibility artifacts in DWI at air-soft tissue borders due to the subcutaneous tumor model. Future studies will have to evaluate the inter- and intraindividual variability of multiparametric imaging in a larger number of subjects, in a variety of different, orthotopic cancers, as well as in cancers of different grades and sizes. Moreover future prospective studies using high field animal MRI may assess the combination of different imaging modalities such as MRI and FDG-PET to improve prediction of long-term tumor outcome.

In conclusion, this study shows successful early treatment monitoring of antiangiogenic therapy using the imaging modalities DWI, DCE-MRI and FDG-PET in a preclinical sarcoma model. DWI, DCE-MRI and FDG-PET are promising imaging techniques for treatment monitoring showing the high percentual change of imaging parameters pre and post treatment and good correlation with final therapeutic success. Thus multiparametric imaging might be promising for monitoring early response to antiangiogenic drugs such as VEGF-A antagonists in future clinical trials.
